# miR-146b Inhibits Glucose Consumption by Targeting *IRS1* Gene in Porcine Primary Adipocytes

**DOI:** 10.3390/ijms19030783

**Published:** 2018-03-09

**Authors:** Yan-Ling Zhu, Ting Chen, Jia-Li Xiong, Di Wu, Qian-Yun Xi, Jun-Yi Luo, Jia-Jie Sun, Yong-Liang Zhang

**Affiliations:** College of Animal Science, Guangdong Provincial Key Laboratory of Animal Nutrition Control, South China Agricultural University, Guangzhou 510642, China; Kerryju27@163.com (Y.-L.Z.); allinchen@scau.edu.cn (T.C.); XJL00LJX@163.com (J.-L.X.); diwu@huskers.unl.edu (D.W.); xqy0228@163.com (Q.-Y.X.); luojunyi@scau.edu.cn (J.-Y.L.)

**Keywords:** miR-146b, glucose consumption, adipocyte, GLUT4, *IRS1*

## Abstract

Adipose tissue plays an important role in energy metabolism. Adipose dysfunction is closely related to obesity and type II diabetes. Glucose uptake is the key step for fat synthesis in adipocyte. miRNAs have been proven to play a crucial role in adipocyte differentiation, adipogenesis and glucose homeostasis. In this paper, we firstly reported that miR-146b decreased glucose consumption by up-regulating miR-146b in a porcine primary adipocyte model, while the inhibitor of endogenous miR-146b rescued the reduction. Then, miR-146b was predicated to target *IRS1* by bioinformatics analysis, and a dual-luciferase reporter assay validated this predication. Western blot analyses indicated both *IRS1* and glucose transporter type 4 (GLUT4) were down-regulated by miR-146b overexpression. Our study demonstrated that miR-146b regulated glucose homeostasis in porcine primary pre-adipocyte by targeting *IRS1*, and provided new understandings on regulations of lipogenesis by miRNAs.

## 1. Introduction

Adipose tissue is no longer considered merely as a storage depot but is also known to participate in immune and inflammation responses [1], blood pressure control [2], thyroid [3], reproductive functions [4], and to make considerable contribution to maintaining glucose homeostatic [5]. Serum glucose is taken up by adipocytes for lipogenesis, and it is a way to keep the concentration of blood glucose at a reasonable level [6,7,8,9]. Insulin is the key hormone in controlling blood glucose, preventing gluconeogenesis in the liver while promoting blood glucose uptake into muscles and adipose tissues via regulating the glucose transporter type 4 (GLUT4) trafficking from intracellular stores to the plasma membrane [10]. In adipose tissues, the absorbed glucose is converted into triglycerides through lipogenesis [11]. GLUT4 is the main glucose transporter in adipocytes, which is found from an intracellular store to the plasma membrane with the insulin trigger [12,13]. GLUT4 contributes a lot to maintain body glucose homeostasis. Both type II diabetes and insulin resistant mice show disruption of GLUT4 expression [14,15]. While GLUT4 is overexpressed, diabetes syndrome is ameliorated in the *db/db* mouse model [16]. The presence of insulin in the extracellular fluids affects metabolic changes via a complicated method [17]. In basal cells, GLUT4 is sequestered in GLUT4 storage vesicles (GSVs) which contain the v-SNARE to form a complex for conducting the GLUT4 translocation to the cell plasma membrane [18]. Only approximately 5% of the GSVs are found on the plasma membrane in the absence of insulin stimulation [19,20]. After insulin binds to the insulin receptor (IR), it promotes the autophosphorylation of a trio of regulatory loop tyrosine residues by activated tyrosine kinase [21]. It phosphorylates IR and leads to the recruitment and Tyr phosphorylation of the insulin receptor substrate (IRS) [10,22,23,24]. The Tyr-phosphorylated IRS1 activates a series of intracellular phosphorylation cascades in motion, resulting in GLUT4 translocation [25]. *IRS1* serves as a docking site for the SH2 domain of PI3K, which activates PI3K and the subsequent phosphorylation of PDK1 and AKT [26]. AKT is activated and phosphorylated through dual Ser/Thr phosphorylation by PDK1 [27]. Activated AKT is attributable to GLUT4 trafficking to the plasma membrane [28,29,30]. Hence, *IRS1* plays a significant role in glucose homeostasis in adipose tissues via affecting GLUT4 translocation in the *IRS1*/GLUT4 signaling pathway.

As a type of non-coding RNAs with approximately twenty-two nucleotides [31], microRNAs(miRNAs) have been involved in many biological processes through their post-transcriptional regulatory influences on genes or transcription factors [32]. Studies have shown that miRNAs are key regulators of the cell cycle [33], adipocyte differentiation [34,35], adipogenesis [36,37], cholesterol and lipid regulation [38,39], insulin signaling and glucose homeostasis [40]. Insulin resistance occurs when the insulin signaling pathway is impaired [10], and it is termed one of the most prevalent metabolic abnormalities [41], which causes obesity and type 2 diabetes (T2D) [10,42]. It has been shown that miRNAs are involved in the insulin signaling pathway [43]; miR-144 directly targets *IRS1* in pancreatic islets isolated from T2D rats [44] and miR-628 benefits burn-induced skeletal muscle atrophy by targeting *IRS1* [45]. *IRS1* and GLUT4 in miR-155 KO mice adipocytes are found to be up-regulated [46].

In our previous study, miR-146a-5p was proved to target the insulin receptors(IR) and, thus, inhibited TNFα-induced adipogenesis in porcine primary adipocytes [47]. The miR-146b was also found to be significantly up-regulated in porcine primary adipocytes after TNFα treatment, but the role of miR-146b remains unclear. In this study, we confirmed that miR-146b decreased glucose consumption by targeting *IRS1* in porcine primary adipocytes involving adipogenesis.

## 2. Results

### 2.1. miR-146b Inhibits Glucose Consumptionin

**Figure 1:**
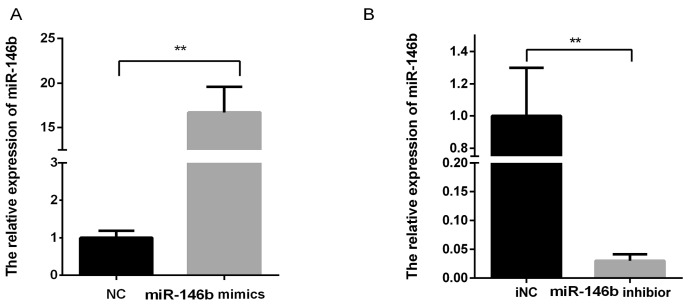
The miR-146b expression levels with transfection of miRNA mimics and inhibitor. Note: miR-146b mimics/NC/inhibitor/iNC were transfected into pre-adipocytes, and cells were collected on day 8 post-induction. After transfection and day 8 post-induction with miR-146b mimics and inhibitor, the expression levels of miR-146b were measured by a qPCR-based method. miRNA mimics (**A**) and inhibitors (**B**) significantly increased and decreased miR-146b levels compared with NC and iNC control, respectively, in adipocytes (*n* = 6, ** *p* < 0.01).

**Figure 2:**
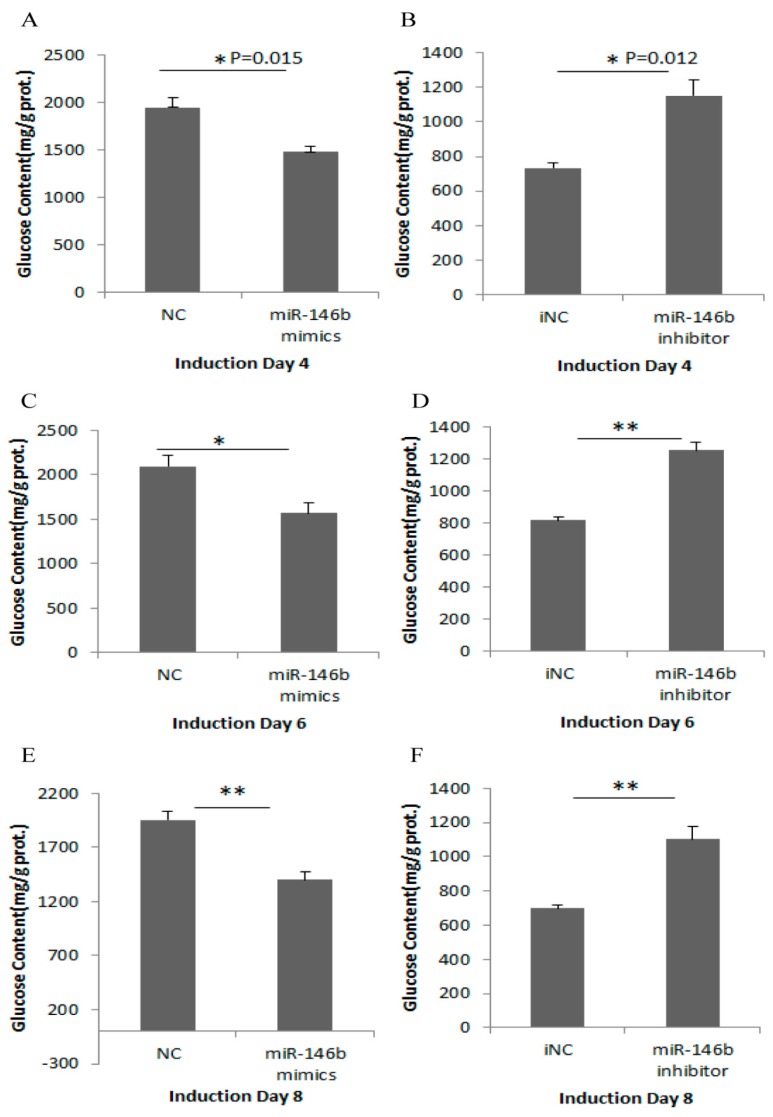
miR-146b inhibits the scavenging effects on glucose in porcine adipocytes. Note: Glucose consumption was determined after transfection of miR-146b mimics/NC or inhibitor/iNC. miR-146b mimics significantly reduced glucose clearance on day 4 (**A**); 6 (**C**) and 8 (**E**) respectively, while inhibitors exerted the opposite results (**B**,**D**,**F**). Data were presented as mean ± SD (*n* = 6, * *p* < 0.05, ** *p* < 0.01).

Porcine primary adipocytes were cultured till the lipid droplets accumulated. miR-146b mimic/NC/inhibitor/iNC were transfected into pre-adipocytes, and cells were collected at days 4, 6 and 8 post-induction. The expression levels of miR-146b were measured by RT-qPCR. The results showed that miRNA mimics significantly increased miR-146b levels, while inhibitors significantly decreased miR-146b expression compared with NC (Figure 1). On days 4, 6, 8 post-transfection, miR-146b mimic obviously reduced scavenging effects on glucose in porcine adipocytes compared with the NC group (Figure 2A,C,E), and this reduction was rescued by the transfection of miR-146b inhibitors (Figure 2B,D,F).

### 2.2. Target Prediction and Pathway Analysis

Target prediction was carried out by the RNAhybrid software, and all the candidates of miR-146b were annotated in KEGG pathway analysis using the DAVID v6.7 online service (https://david.ncifcrf.gov/). The KEGG pathway analysis revealed that the predicted targets of the miR-146b were involved in ten pathways (Table 1), of which adipocytokine signaling pathway is relevant to glucose intake by adipocytes. The predicted targets included *GLUT4* and *IRS1* genes that were related to changes of glucose transport and consumption. Thus, we speculated that miR-146b may affect glucose uptake via these two molecules.

### 2.3. miR-146b Repressed GLUT4 and IRS1 Protein Expression

**Figure 3:**
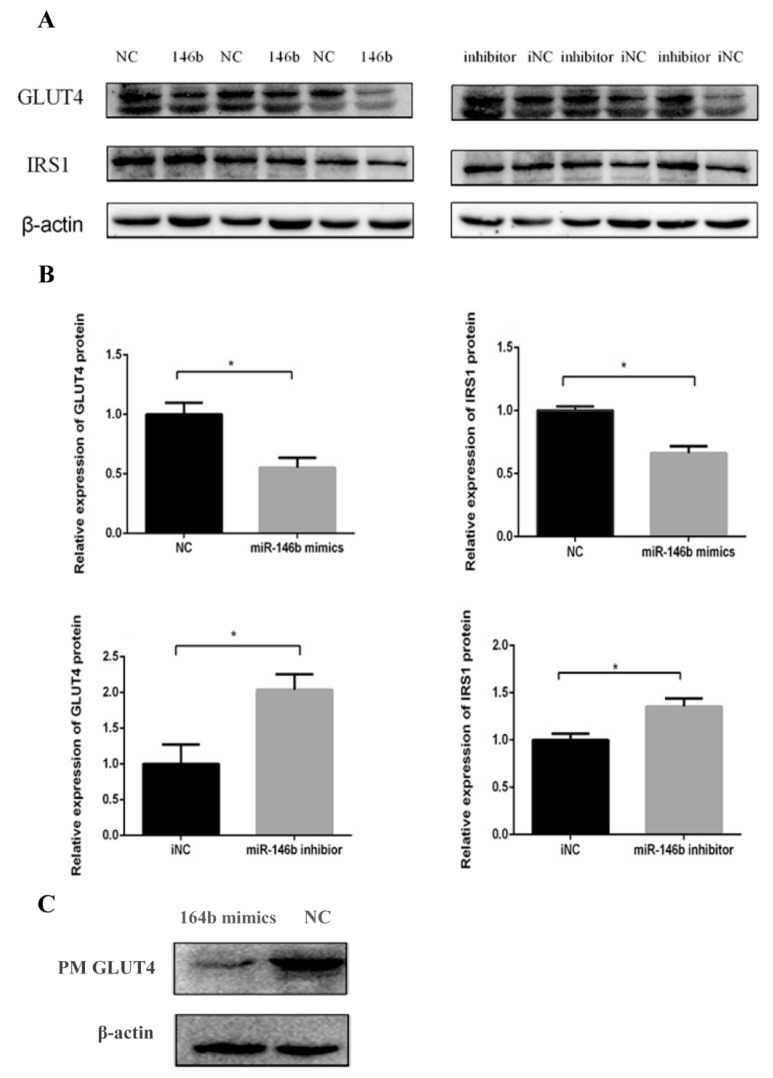
miR-146b repressed GLUT4 and *IRS1* protein expression. (**A**) GLUT4 and *IRS1*protein was down-regulated after miR-146b transfection and this reduction was rescued by the miR-146b inhibitor; (**B**) Data are presented as mean ± SD (* *p* < 0.05, *n* = 6); (**C**) Plasma membrane (PM) proteins were extracted from the differentiated cells and miR-146b mimics reduced the GLUT4 protein expression of the PM.

To explore whether miR-146b could affect the GLUT4 and *IRS1* protein expression levels, we transfected miR-146b mimics, NC, inhibitor and iNC into porcine pre-adipocytes and measured GLUT4 and *IRS1* protein expression at day 8 post-transfection. Western blot analysis showed that miR-146b mimics significantly suppressed both GLUT4 and *IRS1* protein expressions, while the inhibitor rescued the decrease (Figure 3A,B). In addition, previous papers reported that in adipocytes, glucose uptake is dependent on the translocation of GLUT4 from intracellular storage compartments to the plasma membrane (PM) [48,49]. Our results demonstrated that miR-146b reduced GLUT4 translocation to the PM (Figure 3C), which might clarify that miR-146b plays a role in reducing glucose consumption.

### 2.4. Identification of miR-146b Targets by Luciferase Reporter Assay

**Figure 4:**
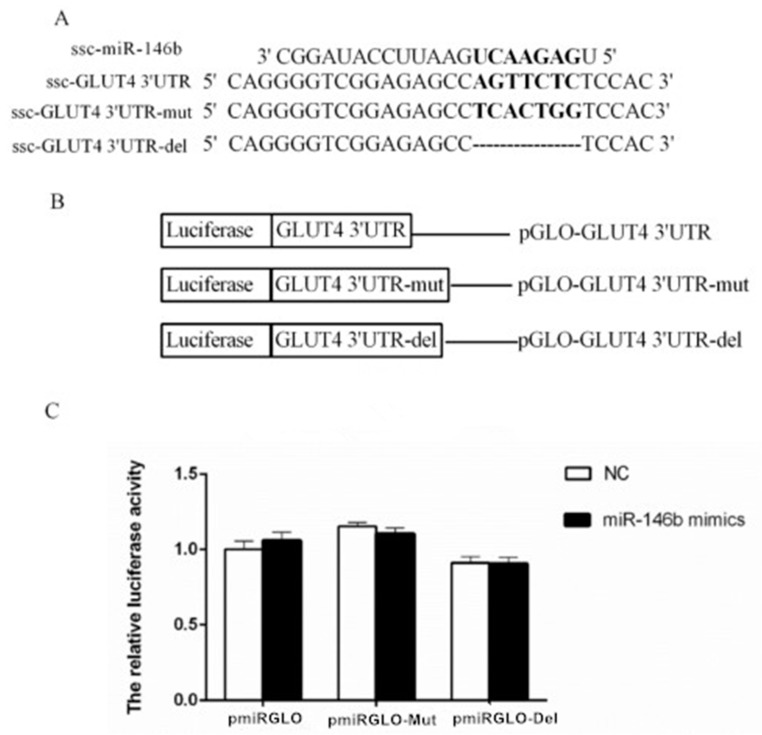
ssc-miR-146b not directly targets porcine GLUT4 by luciferase reporter assay. (**A**) Three 3′-UTR sequences containing normal, mutagenic, and deleted binding sites were inserted downstream of the luciferase reporter. Seven nucleotides of GLUT4 3′-UTR were mutated and deleted to disrupt the binding with miR-146b seed regions; (**B**) Schematic diagram showing dual-luciferase reporter constructs of pig GLUT4 3′-UTR with putative miR-146b binding site; (**C**) Constructed vectors were transfected into Hela cells with miR-146 mimics or NC. The luciferase assay results revealed no significant differences betweenmiR-146b mimics and NC transfected with vectors containing normal GLUT4 3′-UTR, mutant GLUT4 3′-UTR and the deletion (*n* = 8).

**Figure 5:**
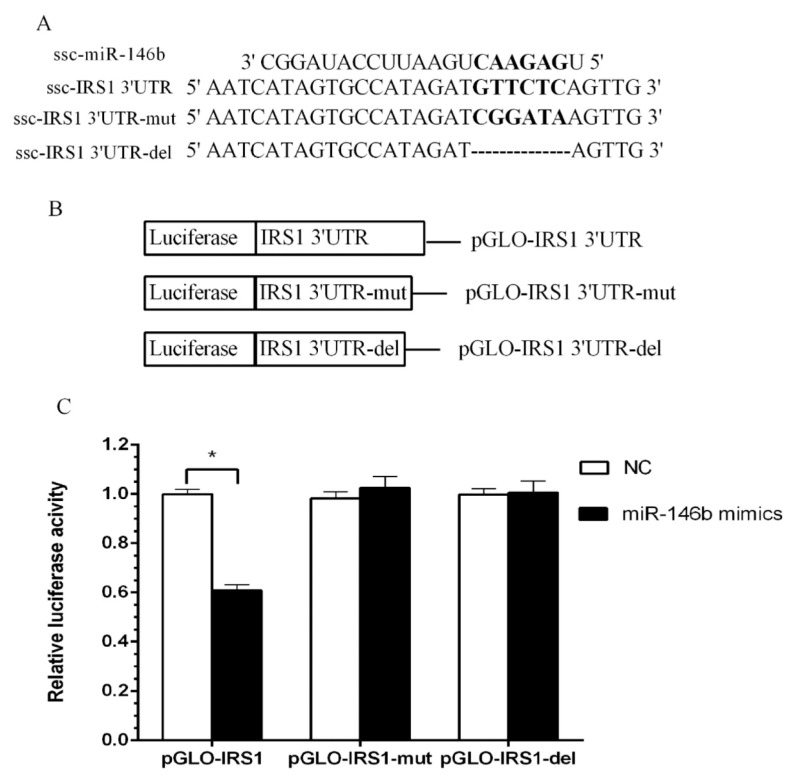
Verification of ssc-miR-146 direct targets of porcine *IRS1* by luciferase reporter assay. (**A**) Three 3′-UTR sequences containing normal, mutagenic, and deleted binding sites were inserted downstream of the luciferase reporter. Six nucleotides of *IRS1* 3′-UTR were mutated or deleted to disrupt the binding with miR-146b seed regions. (**B**) Schematic diagram showing dual-luciferase reporter constructs of porcine *IRS1* 3′-UTR with putative miR-146b binding site; (**C**) Constructed vectors were transfected into Hela cells with miR-146 mimics or NC. The luciferase assay results revealed significant differences between miR-146b mimic and NC groups transfected with vectors containing normal *IRS1* 3′UTR (*n* = 8, * *p* < 0.05).

**Figure 6:**
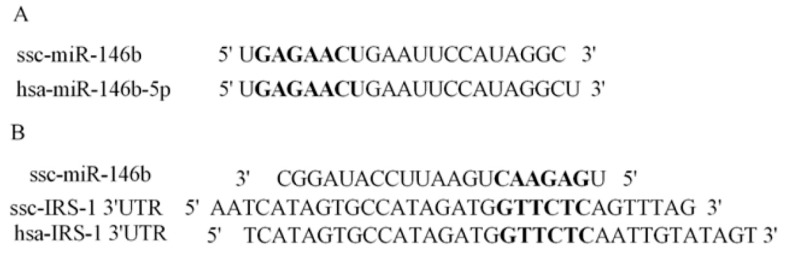
Conserved analysis of miR-146b and its target gene across species.(**A**) The bold letter showed that porcine miR-146b shared the same sequences of seed region with human miR-146b; (**B**) The bold pointed out that 3′UTR of human *IRS1* mRNA is in accordance with ssc-miR-146b binding site.

To validate the targeted relationship between miR-146b and 3′UTR of porcine *GLUT4* (Figure 4A) and *IRS1* (Figure 5A) genes, the wild-type (pGLO-3′UTR), mutant and deletion (pGLO-3′UTR-mut and pGLO-3′UTR-del) plasmid (Figure 4B and Figure 5B) were co-transfected with a miR-146b mimic into Hela cells. At 48 h post-transfection, the luciferase activity of each group was assayed, and for target *IRS1* gene, the miR-146b mimics group showed lower luciferase activity when compared with the NC group (Figure 4C and Figure 5C). The reduction was rescued both by mutation and deletion of the seed sequence. Intriguingly, miR-146b was found to be highly similar between pigs and humans (Figure 6A). The 3′UTR of pigs or human *IRS1* mRNA contains a binding site which perfectly matched the seed region of miR-146b (Figure 6B).

### 2.5. miR-146bend Base Mutation Changes miR-146b Targeting on IRS1 by Luciferase Reporter Assay

**Figure 7:**
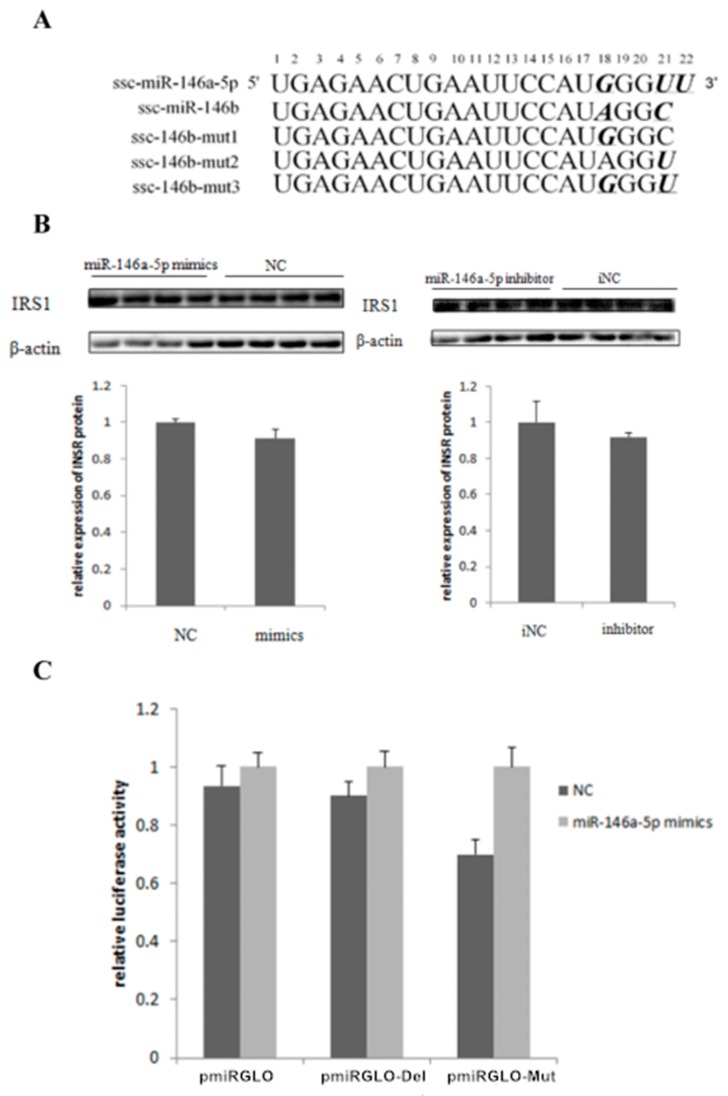
miR-146a-5p did not target *IRS1* gene (**A**) miR-146a-5p is different from miR-146b at the 18th and the 21th base, and there is one more base at the end of miR-146a-5p. We therefore synthesized three mutants. For mut1, the 18th base A was replaced by G; for mut2, the 21th base C was replaced by U; for mut3, both the 18th and the 21th base were replaced according to the sequence the miR-146a-5p. (**B**) Western blot and gray-scale scanning analyses of *IRS1* after transfection of miR-146a-5p mimics/NC/inhibitor/iNC (*n* = 4); (**C**) pmirGLO dual-luciferase reporter vectors analysis between miR-146a-5p and the 3′UTR sequences of *IRS1* gene.

**Figure 8:**
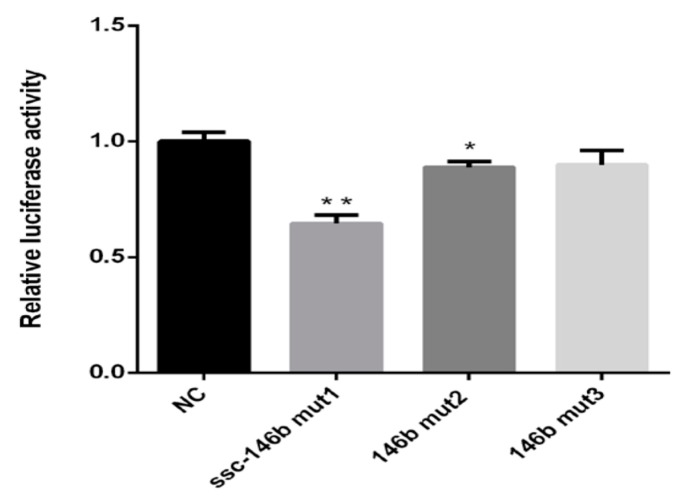
Analysis of end base mutation changes of miR-146b targeting on *IRS1* by luciferasereporter assay (*n* = 8, * *p* < 0.05, ** *p* < 0.01).

Since miR-146a-5p and miR-146b share the same seed region (Figure 7A)and only few end base mutations exist, three miR-146 mutants were synthesized to test the contribution of the end base on determination of miR-146b targets(Figure 7A). A targeted relationship between miR-146a-5p and the *IRS1* gene was not detected in this paper (Figure 7A,C). The three mutants were co-transfected with the wild-type (pGLO-IRS1-3′-UTR) plasmid into Hela cells. At 48 h post-transfection, the luciferase activity of the wild-type *IRS1* reporter was significantly reduced by miR-146b mut1 compared with the NC. The miR-146b mut2 group also elicited similar results but the extent of the reduction is less than the ssc-miR-146b mut1 (Figure 8). The results showed that mutant 1 and mutant 2 still targeted 3′-UTR of *IRS1* while mutant 3 failed to target *IRS1*, which indicated that the 18th and 21st base of miR-146b play a significant role in ssc-miR-146b targeting *IRS1*.

## 3. Discussion

Adipose tissue mainly functions as a storage site for storing glucose and transforming into adipose. The profound effect of adipocytes on glucose balance is mediated by various mechanisms, which mostly performs in adipose tissue. Too much fat in the body exerts obesity and too little fat would exert lipodystrophy [8]. Obesity has become a worldwide problem, especially in Western countries. It can increase the risk of numerous diseases, particularly heart disease, type II diabetes, osteoarthritis and some types of cancer [50]. Thus, fully comprehending the mechanism of insulin signaling pathways and other biological processes in the adipose tissue is beneficial for exploring a potential target of obesity treatment.

Adipocytes uptake serum glucose to reserve energy as triglycerol and this process requires GLUT4 to transport the glucose from extracellular to intracellular locations [51,52]. GLUT4 is identified as the main glucose transporter in adipocytes [12,13]. After food consumption, glucose in the serum increases and GLUT4 is transported from an intracellular location in the adipocyte to the cell surface to uptake glucose for adipogenesis [53]. In the adipocyte, insulin signaling pathways control glucose transport via the *IRS1*/PI3K/GLUT4 signaling pathway. After insulin binds to IR, activated IR phosphorylates *IRS1* [47]. Tyrosine-phosphorylated *IRS1*, recruits PI3K and later activates the GLUT4 translocation [48]. Therefore, reduced *IRS1* expression could repress the GLUT4 translocation in adipocytes and also skeletal muscles, which results in the decrease of glucose uptake. Such phenomena always accompany insulin resistance and diabetes [10,54,55,56,57].

The miR-146 family of microRNAs consists of two members, miR-146a and miR-146b. These two miRNAs are located on different chromosomes and conduct different functions in the body. Additionally, many studies have illustrated that miR-146a and miR-146b target different genes in various cases. Studies of miR-146a and miR-146b mostly focus on inflammation [58,59,60,61,62] but the biological functions of the miR-146 family in adipose tissue remains largely unknown. In our previous study, the expression level of miR-146b was dramatically increased by 13.62-fold after TNFα-induced adipogenesis according to a microarray assay analysis [47]. It is reasonable to suppose that miR-146b plays a vital role in adipocyte. Additionally, whether its target gene is identical to miR-146a-5p is worth exploring since miR-146b shares the same seed with miR-146a-5p.

In this study, we examined the glucose consumption of adipocytes on days 4, 6 and 8. The glucose consumption was decreased by miR-146b mimics, and it was rescued by a miR-146b inhibitor. GLUT4 total protein in adipocyte was also significantly reduced by miR-146b mimics, and it was also rescued by the inhibitor. Therefore, we speculated that the reduction of the glucose uptake capability of adipocytes was related to GLUT4. In addition, PM GLUT4 protein was also reduced by miR-146b mimics. Thus, we speculated that miR-146b inhibiting GLUT4 translocation would be the reason for the reduced glucose consumption caused by miR-146b. However, luciferase reporter assays showed that ssc-miR-146b did not directly target porcine GLUT4. We supposed there might be other molecules involved in this process.

To further validate the mechanism of miR-146b regulating adipocyte glucose consumption, we examined the protein level of *IRS1*, since *IRS1* is significantly related to GLUT4 translocation via insulin signaling. We found that miR-146b mimics suppressed *IRS1* protein expression significantly, while the inhibitor rescued this decrease. Then, we carried out a luciferase reporter assay and verified that miR-146 directly targeted *IRS1*. Thus, we confirmed that miR-146 represses adipocyte glucose uptake via the *IRS1*/GLUT4 pathway by targeting *IRS1*.

As for the pig miR-146 family, miR-146a-5p and miR-146b shared the same seed region while only the two nucleotides on the 3′end of the mature strand are different. In our previous study, miR-146a-5p was proved to target insulin receptor (IR) and thus inhibited lipogenesis in porcine primary adipocytes [47]. In this paper, miR-146b inhibited glucose consumption by targeting *IRS1* instead of IR. To determine why miR-146a-5p and miR-146b target different genes although they share the same seed region, we synthesized three mutants of miR-146b; the end base was replaced to be in accordance with the miR-146a-5p sequence. We found out that only when the 18th and 21st base of ssc-miR-146b were replaced, the target relationship between miR-146b and *IRS1* would be lost according to the luciferase reporter assay. It is possible that the regulatory functions of miR-146a-5p and miR-146b would be decided by the sequence outside of the seed region. Because of the stabilization of the miRNA-mRNA complex, which is formed during miRNA exerting its biological function, regulatory functions could be more probably affected by the 3′ end and not sequences in the seed of the mature miRNA [63], which may explain the result that miR-146a-5p and miR-146b target different genes.

Therefore, our study is the first to illustrate that miR-146b regulates glucose homeostasis in porcine primary pre-adipocytes by targeting *IRS1* and it could be a potential target of type II diabetes in the future.

## 4. Materials and Methods

### 4.1. Ethics Statement

All of the animal experiments were performed under the instructions of Guangdong Province on the Review of Welfare and Ethics of Laboratory Animals approved by the Guangdong Province Administration Office of Laboratory Animals (GPAOLA). All animal procedures were carried out with the guidelines of the protocol (SCAU-AEC-2010-0416) approved by the Animal Ethics Committee of South China Agricultural University.

### 4.2. Sample Collection and Culture of Porcinepre-Adipocytes

Subcutaneous fat tissues from a 7-day-old piglet was isolated aseptically and transferred to Dulbecco’s modified essential medium–F12 nutrient mixture (GIBCO, New York, NY, USA). After removing the visible connective tissues, the adipose tissue was cut into small cubes of about 1 mm^3^, and the subcutaneous pre-adipocytes were obtained as described in our previous study [47]. Minced tissue was transferred into a Carlsberg’s flask, digested in 0.2% type-II collagenase (1 mg/mL, GIBCO) for 2 h at 37 °C, and then filtered through a 150 μm mesh. Cells in the filtrate were centrifuged at 500 *g* for 10 min, and erythrocytes were lysed using erythrocyte lysis buffer (0.154 MNH_4_Cl, 10 mM KHCO_3_ and 0.1 mM EDTA). After filtering through a 40 μm mesh, cells were rinsed with F12 and centrifuged at 1500 *g* for 5 min. The pre-adipocytes were collected and plated in growth medium. The pre-adipocytes were cultured in 6-well plates and induced to mature adipocytes with induction medium(10% FBS, F12, 50 μM oleic acid, 0.5 M Moctoic acid, 50 nM insulin, 50 nM dexamethasone, and these reagents were purchased from Sigma, Ronkonkoma, NY, USA).

### 4.3. Target Prediction and Pathway Analysis

The 3′-untranslated region (UTR) sequences of porcine transcripts in the whole genome were obtained from Ensembl genome browser 80 (sscorfa10.2, www.ensembl.org/Sus_scrofa/). Porcine miR-146b sequences were downloaded from miRBase release 21 (www.mirbase.org), and RNAhybrid software (BGI, Shenzhen, China) was used to analyze miRNA targets by using its own algorithm. Our prediction was restricted to a perfect match of the seed region (2–7 bases of the miRNA 5′ end; G:U matches were permitted), due to the importance of the seed sequence for miRNA-mRNA binding. In addition, the Database for Annotation, Visualization and Integrated Discovery (DAVID) v6.7 online service (http://david.abcc.ncifcrf.gov/) was used for Gene Ontology (GO) and Kyoto Encyclopedia of Genes and Genomes (KEGG) pathway analysis based on the potential targets of miR-146b.

### 4.4. Transfection of miR-146b Mimics and miR-146b Inhibitor

miR-146b mimics, miR-146b inhibitors, negative control (NC and iNC) were acquired from Shanghai GenePharma Co. (Shanghai, China). Pre-adipocytes at 80–90% confluency were used for transfections, and miRNA mimics or inhibitors of 100 pmol was prepared in each well. NC and iNC were used as negative controls for the miR-146b mimics and miR-146b inhibitors, respectively. Transfection was performed using Lipofectamine 2000 (Invitrogen, Carlsbad, CA, USA). After 6 hours of the transfection, the cell culture medium was replaced with induced medium. On the 4th, 6th and 8th day of induction, the supernatants (mimics group, NC group, inhibitor group and iNC group) were collected to conduct the glucose consumption assays and the cells induced to 8th day were conducted for Western blot of GLUT4 and *IRS1*.

### 4.5. RNA Extraction and Real-Time PCR for miR-146b

Total RNA was extracted from differentiated adipocytes using TRIzol reagent (Invitrogen™, Carlsbad, CA, USA), and RNA quality was determined by the NanoDrop 2000(Thermo Fisher Scientific, MA, USA). Total RNA (2 μg) was reverse-transcribed using M-MLV reverse transcriptase (Promega, Madison, WI, USA) with a specific hairpin primer for miR-146b (5′-CTCAACTGGTGTCGTGGAGTCGGCAATTCAGTTGAGGCCTAT-3′). Real-time PCR was performed with Bio-Rad CFX-96 thermocycler (Bio-Rad, Hercules, CA, USA) using 2× SYBR Green PCR Master Mix (Promega, Madison, WI, USA) with a miRNA-specific forward primer (5′-CAGTGAGAACTGAATTCCATAGGC-3′) and a universal reverse primer (5′-ATCCAGTGCGTGTCGTGGA-3′). The PCR reaction (20 μL) consisted of 2μL of cDNA, 1.5 μM of each primer, 10 μL of 2× SYBR Green PCR Master Mix and distilled water. The reactions were processed for 5 min at 95 °C, followed by 40 cycles of 15 s at 95 °C, 15 s at 58 °C, and 40 s at 72 °C. miRNA expression level was normalized to that of the internal control U6 (sense: TGCTTCGGCAGCACATATAC, antisense: TTCACGAATTTGCGTGTCAT) in each sample by the 2^−ΔΔ*C*t^ method. Statistical differences between treatment and control groups were determined using Student’s *t*-test at *p* < 0.05.

### 4.6. Glucose Consumption Assay

Glucose consumption measurement was performed on used medium samples. During the differentiation of porcine pre-adipocytes, the supernatant was collected on day 4, 6 and 8 to 1.5 mL centrifuge tubes for testing. Glucose consumption was assayed using the Glucose Assay Kit (BioSino, Beijing, China)based on the glucose oxidase method [64]. Total protein detected by the bicinchoninic acid (BCA) assay (Thermo Fisher Scientific, Waltham, MA, USA) was used for normalization of glucose content.

### 4.7. Protein Extraction and Western Blot 

Total proteins were extracted using radio immunoprecipitation assay (RIPA) buffer with protease inhibitors (Boston Bio Products, Boston, MA, USA). Plasma membrane (PM) proteins were extracted using the Membrane Protein Extraction Kit (BestBio, Shanghai, China). Protein levels were quantified by the BCA protein assay. Total protein (25 μg) was loaded onto a 10% SDS-PAGE gel, separated by electrophoresis and transferred onto a polyvinylidene difluoride (PVDF) membrane. Blots were blocked with 6% skim milk and incubated overnight at 4 °C with primary antibody against GLUT4 (Abcam, Cambridge, UK)and *IRS1* (Proteintech Group, Chicago, IL, USA), followed by incubation with secondary antibody for 1 h at room temperature and measured with a Fluor Chem M (ProteinSimple, Santa Clara, CA, USA). Protein expression was normalized by detection of β-actin (Abcam, Cambridge, UK). Duplicate experiments were conducted for all primary adipocyte proteins. The data were analyzed by Image J software (National Institutes of Health, Bethesda, MD, USA) and expressed as fold-change relative to the control group after normalizing against β-actin. Statistical differences between treatment and control groups were determined using Student’s *t*-test at *p* < 0.05.

### 4.8. Plasmid Construction

The 3′-UTR sequences of porcine transcripts in the whole genome were obtained from NCBI (http://www.ncbi.nlm.nih.gov/). The 3′-UTR of *IRS1* (Accession No. NM_001244489.1) contains the highly conserved binding sites for the miR-146b. The 3′-UTR sequence was inserted into pmirGLO Vector (Promega) with *Xho*I and *Xba*I double digestion to construct recombinant Dual-Luciferase reporter vectors, named as pGLO-IRS1-3′-UTR, pGLO-IRS1-3′-UTR-mut and pGLO-IRS1-3UTR-del. Meanwhile, mutagenic and deleted *IRS1* 3-′UTR reporter vector swere constructed with seven exchanged nucleotides or a deleted target site via DNA synthesis (Sangon Biotech, Shanghai, China).

### 4.9. Dual-Luciferase Reporter Assay

Hela cells were seeded at density of 3 × 10^4^ cells per well in 96-well plates. When the cells reached 60–70% confluency, wild-type (pGLO-IRS1-3′-UTR), mutant (pGLO-IRS1-3′-UTR-mut and pGLO-IRS1-3′-UTR-del) plasmids were co-transfected with miR-146b mimics or negative control (NC) into Hela cells. Lipofectamine 2000 (Invitrogen) was used for mediating the transfection. Cells were collected at 48 h post-transfection, and the luciferase assays were performed with the Dual-Luciferase reporter assay system (Promega). The luciferase activities were normalized by renilla activity. Statistical differences between treatment and control groups were determined using Student’s *t*-test at *p* < 0.05.

## Figures and Tables

**Table 1:** KEGG pathway analysis of candidates predicted by RNAhybrid software.

Pathway Term	Genes	*p*-Value
Adipocytokine signaling pathway	*CPT1*, *FACS*, *IRS*, *AMPK*, *GLUT4*, *LEPR*	2.7 × 10^−3^
T cell receptor signaling pathway	*CD4/8*, *CD3y*, *Rho*, *Cdb42*, *NFAT*, *Ras*, *ICOS*	3.2 × 10^−3^
Phagosome	*MHCII*, *TLR4*, *Dynein*, *SRB1*, *TUBB*, *vATPase*	3.8 × 10^−3^
Focal Adhesion	*ECM*, *ITGA*, *ITGB*, *RhoA*, *MLC*, *Caveolin*, *Vinculin*	4.8 × 10^−2^
Regulation of actin cytoskeleton	*ITG*, *Ras*, *Rho*, *VCL*, *PI4P5K*, *MLC*, *Arp2/3*, *WAVE2*	1.8 × 10^−2^
Bacterial invasion of epithelial cells	*WAVE*, *Arp2/3*, *RhoA*, *Vinculin*, *Caveollin*	2.0 × 10^−2^
AMPK signaling pathway	*Ob-Rb*, *AMPK*, *GLUT4*, *IRS1*, *Rab*, *CPT1*	2.2 × 10^−2^
FoxO signaling pathway	*Smad4*, *AMPK*, *GLUT4*, *IRS*, *Ras*, *ATM*	3.5 × 10^−2^
Neurotrophin Signaling Pathway	*NT4*, *IRS1*, *Ras*, *RhoA*, *p53*	7.7 × 10^−2^
